# Impact of a Geriatric Intervention to Improve Screening and Management of Undernutrition in Older Patients Undergoing Surgery for Colorectal Cancer: Results of the ANC Stepped-Wedge Trial

**DOI:** 10.3390/nu13072347

**Published:** 2021-07-09

**Authors:** Thomas Gilbert, Lorraine Bernard, Marine Alexandre, Sylvie Bin-Dorel, Laurent Villeneuve, Evelyne Decullier, Marc Bonnefoy, ANC Working Group

**Affiliations:** 1 Geriatric Medicine Department, Hospices Civils de Lyon, Groupement Hospitalier Sud, CEDEX, 69495 Pierre-Bénite, France; thomas.gilbert@chu-lyon.fr; 2 Research on Healthcare Professionals and Performance RESHAPE, Inserm U1290, Université Claude Bernard Lyon 1, 69008 Lyon, France; 3 Public Health Department, Epidemiology and Clinical Research Unit, Hospices Civils de Lyon, 69002 Lyon, France; lorraine.bernard01@chu-lyon.fr (L.B.); marine.alexandre@chu-lyon.fr (M.A.); sylvie.bin@chu-lyon.fr (S.B.-D.); laurent.villeneuve@chu-lyon.fr (L.V.); evelyne.decullier@chu-lyon.fr (E.D.); 4 CarMeN Laboratory, Inserm U1060, INRA U1397, Université Claude Bernard Lyon 1, 69100 Oullins, France

**Keywords:** neoplasms, preoperative care/methods, enteral nutrition, aged

## Abstract

Almost two in three patients who are aged 75 years and older and scheduled for surgery for colorectal cancer (CRC) are undernourished. Despite evidence that perioperative nutritional management can improve patients outcomes, international guidelines are still insufficiently applied in current practice. In this stepped-wedge cluster-randomized study of five surgical hospitals, we included 147 patients aged 70 years or older with scheduled abdominal surgery for CRC between October 2013 and December 2016. In the intervention condition, an outreach team comprising a geriatrician and a dietician visited patients and staff in surgical wards to assist with the correct application of guidelines. Evaluation, diagnosis, and prescription (according to nutritional status) were considered appropriate and strictly consistent with guidelines in 39.2% of patients in the intervention group compared to only 1.4% in the control group (*p* = 0.0002). Prescription of oral nutritional supplements during the perioperative period was significantly improved (41.9% vs. 4.1%; *p* < 0.0001). However, there were no benefits of the intervention on surgical complications or adverse events. A possible benefit of hospital stay reduction will need to be confirmed in further studies. This study highlights the importance of the implementation of quality improvement interventions into current practice for the perioperative nutritional management of older patients with CRC.

## 1. Introduction

Colorectal cancer (CRC) is the third most frequently diagnosed type of cancer worldwide [1]. The incidence of CRC increases with age, and the risks of mortality and morbidity are significantly increased after 70 years [2]. In older patients, the prognosis can be strongly impacted by competing comorbidities and frailty syndromes [3], among which undernutrition plays a major part [4,5,6,7]. By way of illustration, the G8 screening tool for assessing the prognosis of aged cancer patients is mainly based on their nutritional status [8].

While undernutrition can concern 20% to 80% of older patients with cancer [9,10,11], the prevalence of undernutrition has been estimated at around 40% in aged patients (>70 years) with advanced colorectal cancer [12]. The presence of cancer can also lead to cachexia, characterized by continuous weight loss, the consequence of abnormal metabolic activity and anorexia as well as altered immune function [13]. This might also trigger sarcopenia, which will affect patients’ physical recovery after surgery due to decreased muscle strength and function [11].

In addition, it could be shown that undernutrition is a risk factor of postoperative mortality and complications, such as infection or healing problems, increased length of stay, or altered quality of life [6,7,14,15,16]. In particular, weight loss >10–15% within six months prior to surgery [2] and serum albumin levels <30 g/L [17] are independent factors of mortality and morbidity.

Meanwhile, there is evidence that optimizing nutritional management in the perioperative period can improve patient outcomes, especially as part of multimodal prehabilitation interventions [9,14,18,19,20]. A recent meta-analysis highlighted that nutrition is a key component of prehabilitation and that multimodal interventions should, at minimum, comprise both nutrition and exercise components [14]. This meta-analysis showed that nutrition (with or without exercise) is efficient at reducing the length of stay by two days after colorectal surgery [14]. The results also suggested that prehabilitation programs with nutrition as the main component and exercise as an adjunct component are beneficial to improve clinical and functional recovery after surgery, although the heterogeneity of the included studies prevented firm conclusions [14].

International guidelines have been established for the perioperative screening and management of undernutrition in older patients with cancer [21,22,23,24]. Considering that cancer-related undernutrition is often overlooked by clinicians as well as patients and their families, the ESPEN study group sent out a call for action to emphasize the importance of putting the guidelines into full practice [9]. Key recommendations include screening all patients with cancer for nutritional risk, regardless of BMI and weight history; increasing nutrition assessments (including measures of anorexia, body composition, inflammatory biomarkers, resting energy expenditure, and physical function), and using individualized plans to increase nutritional intake, decrease inflammation and hypermetabolic stress, and promote physical activity [9].

Despite proven individual benefits, these guidelines for the perioperative period are still insufficiently applied in everyday practice [9]. Nutritional assessments are often limited to the measurement of weight and body mass index, and elements such as weight loss or anorexia remain rarely assessed. Furthermore, nutritional support may not be prescribed systematically for undernourished older patients with CRC, especially in the preoperative period. This could be due to a lack of awareness or insufficiently structured pathways and management procedures in a context of constraints in resources or time due to surgical targets [25].

Therefore, we hypothesize that setting up a specific process of screening and management of undernutrition with the intervention of a dedicated geriatric outreach team might improve adherence to guidelines and the outcomes of older patients undergoing surgery for colorectal cancer.

## 2. Materials and Methods

### 2.1. Study Design and Setting

The ANC study (“Age, Nutrition, Cancer”; ClinicalTrials.gov reference number: NCT0208452) was an open-label prospective multicenter cluster-randomized trial with a stepped-wedge design, as previously described [26]. Five surgical departments (two university hospitals, two private clinics, and one cancer reference center) with regular activity in CRC surgery were enrolled in the study. All centers (clusters) started by including patients in the control phase (management as usual), and the implementation of the intervention was rolled out sequentially in each center every six months until all centers benefited from the intervention phase for at least six months. The time at which each cluster switched from the control condition to intervention was randomized.

### 2.2. Participants

Eligible subjects were patients aged 70 or older, admitted for scheduled surgery of CRC (with or without concomitant metastases), covered by national health insurance, who had given their informed consent to participate. We excluded patients hospitalized for emergency surgical resection as well as patients under a legal safeguarding measure. Patients were included from October 2013 to December 2016.

### 2.3. Study Intervention

During the control phase, surgical centers continued to manage patients in accordance with their current practice. The screening and management of undernutrition were appraised retrospectively by the study team based on the medical records of the included patients.

**Figure 1:**
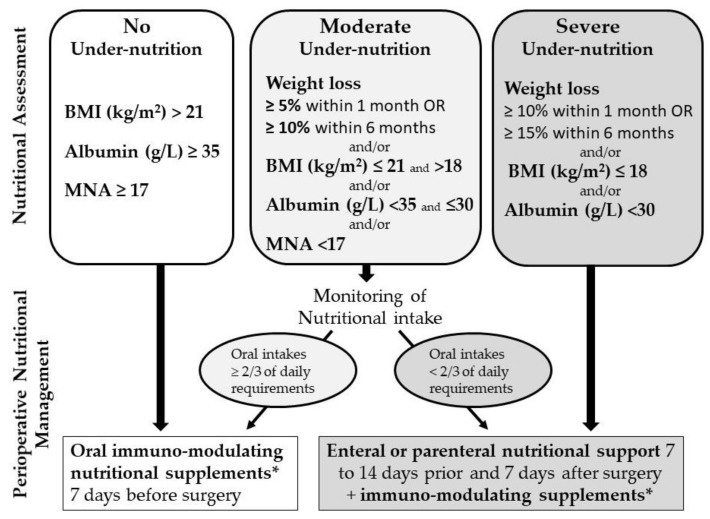
Perioperative undernutrition screening and management for CRC surgery. Proposed algorithm during the ANC study, based on guidelines from ESPEN [21,23,24] and French SFAR/SFNEP guidelines [22]. * Arginine, omega-3 fatty acids, and nucleotides: 3 supplements/day 7 days before surgery (1000 Kcal/d) and 7 days after surgery for undernourished patients (1500 Kcal/d).

In the intervention phase, an outreach geriatric team visited each center to provide training and advice to the health professionals focused on nutritional screening and management of undernutrition in accordance with prevailing guidelines [23]. This was done through scheduled meetings with the staff, regular visits to the wards, and the dissemination of written, informative documents to both patients and professionals. In addition, a systematic multi-professional assessment was carried out for all patients in the intervention phase, including at least a geriatrician and a dietician. The intervention was based on recommendations from the French government [22] as well as on the 2006 guidelines from the European Society for Clinical Nutrition and Metabolism (ESPEN) [23], which have since been actualized in 2017 [24] after the intervention and data collection took place. The proposed patient management algorithm is shown in Figure 1. A more detailed description of the intervention has been published previously [26].

### 2.4. Data Collection

Data were collected for both groups from medical records by a clinical research assistant and reported in an electronic Case Report Form (CRF).

Baseline characteristics (at least 7 days before surgery) considered for each patient, irrespective of the study phase, included: age, sex, weight, height, body mass index (BMI), weight loss (with rate over time), Mini-Nutritional Assessment (MNA) [27], level of comorbidity (assessed using the geriatric Cumulative Illness Rating Scale (CIRS-G [28]), and number of daily medications. A blood sample was taken for hemoglobin (Hb), serum albumin, and pre-albumin, as well as C-reactive protein (CRP). An evaluation of physical performance was performed on both groups using the short physical performance battery (SPPB) [29] and the measurement of handgrip strength. We assessed the presence of fatigue using an analogical visual scale (AVS) [30] and mood using the mini-GDS (score on 4) and the GDS 15 (if mini-GDS score > 2) [31].

Appetite was appraised using a modified version of the AC/S-12 subscale of the FAACT questionnaire (Functional Assessment of Anorexia/Cachexia Therapy) [32]. Where possible, the presence of cachexia was assessed on the basis of the definition by Evans et al. [13]. That is, a BMI <20 Kg/m^2^ or weight loss equivalent to more than 5% in 12 months, associated with three or more of the following criteria: decreased muscle strength (handgrip strength <30 Kg for men or <20 Kg for women, short physical performance battery score <7, fatigue (score ≤ 3 on fatigue AVS), anorexia (as declared in the medical records), anemia (Hb < 12 g/L), serum albumin <32 g/L, or increased inflammatory markers (C-reactive protein > 5 mg/L). Patients with missing data and without undernutrition were considered to be without cachexia.

### 2.5. Primary Outcome

The primary outcome of the study was the level of consistency of the perioperative nutritional management of patients with the 2006 ESPEN guidelines (which prevailed at the time of the study) [23]. To be considered in accordance with the guidelines, patients had to fulfill three criteria: presence of a complete nutritional assessment, accurate evaluation of nutritional status, and prescription adapted to nutritional status.

Nutritional assessment was considered complete in the presence of the following: calculation of BMI, evaluation of recent weight loss, blood sampling for albumin assay at least seven days prior to surgery, and MNA at least seven days prior to surgery.

Accurate evaluation of the nutritional status meant moderate (severe) undernutrition, defined as either albumin <35 g/L (<30), BMI <21 Kg/m^2^ (<18), weight loss of at least 5% (10%) in one month or 10% (15%) in six months, or MNA <17. In addition, patients with moderate undernutrition and oral intake <2/3 of recommended daily requirements were classified as severely undernourished. The study team reassessed the nutritional status of all patients according to these criteria, irrespective of the study period, based on medical records and study documents. Any discrepancy with the diagnosis made on site by the team in charge of the patient was recorded.

Prescriptions considered adapted to nutritional status were: three or more immune-modulating supplements prescribed at least seven days prior to surgery in the absence of undernutrition or prescribed for seven days before and seven days after surgery in the case of moderate undernutrition; artificial nutrition proposal for 7–14 days before surgery and seven days after in the case of severe undernutrition or moderate undernutrition with the presence of anorexia.

### 2.6. Secondary Outcomes

Prevalence of moderate and severe undernutrition in this population was sought as well as the proportion of patients presenting characteristics of cachexia [13] (see above).

The potential effects of the intervention on the rate and type of postoperative complications were analyzed up to 30 days after surgery. These were graded from I to V in terms of severity, referring to the Common Terminology Criteria for Adverse Events (CTCAE) of the National Cancer Institute, version 4.0., and the Clavien–Dindo classification [33].

Of note, due to missing data, we were not able to report on the consumption of immune-modulating ONS or the change in the level of independence prior to and after surgery, as initially envisaged [26].

### 2.7. Statistical Analysis

Descriptive data were reported as median (range), number of patients, and percentages. Categorical variables were analyzed using the chi-squared test or Fisher’s exact test, as appropriate. Continuous variables were analyzed using Student’s *t*-test or the Wilcoxon test for variables that did not satisfy normality. The application of Fisher’s test or Wilcoxon’s test is specified by an asterisk (“*”) next to the *p*-value.

Analysis of the primary outcome: Mixed-effects logistic regression with a random intercept was used to analyze the level of consistency of perioperative nutritional management before and after the intervention. Statistical significance was defined as *p* < 0.05.

All statistical analyses were performed under the intention-to-treat principle using SAS 9.3 (Institute Inc., Cary, NC, USA).

### 2.8. Ethics

The study was approved by the institutional review board (CPP: Comité de Suivi des Personnes) on 21 November 2012 and the CNIL (Comité National Informatique et Libertés) on the 28 February 2013 (DR 2013-083). Investigators were advised to offer participation to each consecutive patient eligible for inclusion. All patients agreed to participate.

## 3. Results

### 3.1. Participants

**Figure 2:**
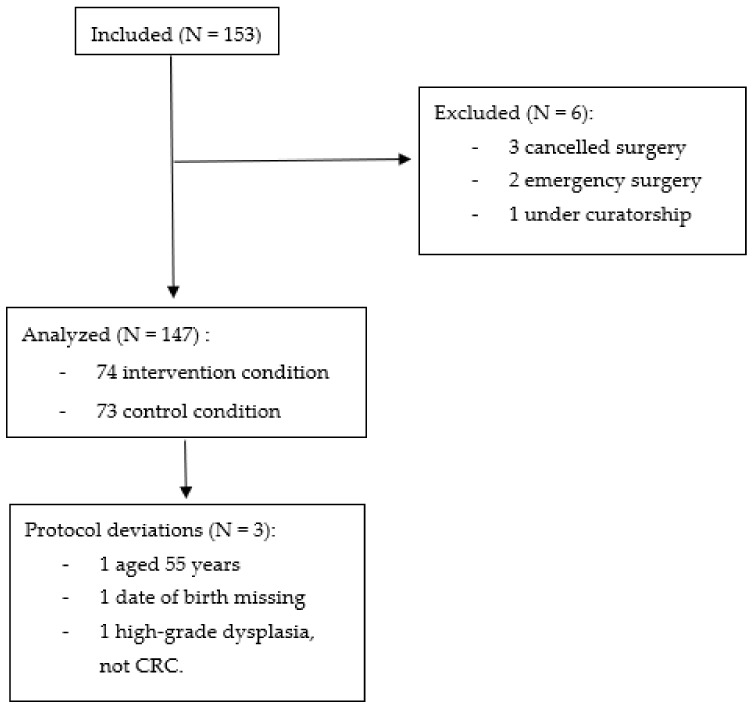
Study flow chart.

Figure 2 and Table 1 present the study flow chart and details of the included patients at each step. Among the 153 patients included, 6 were excluded from the analysis (Figure 2). Finally, we considered 147 patients for the intention-to-treat analysis, among which 74 patients were in the intervention condition and 73 were in the control condition.

Baseline characteristics of included patients are presented in Table 2. Sixty-one patients (62%) were classified as undernourished (severe or moderate) overall. There was a larger proportion of undernourished patients in the control condition compared to the intervention condition. Cachexia was present in less than 6% of patients (Table 2). Cancer localization and surgery type are presented in Table S1.

### 3.2. Results on the Primary Outcome

Nutritional management was strictly in accordance with 2006 ESPEN guidelines [23] in 30 patients (20.4%) out of the 147. Using mixed models to account for the stepped-wedge design, we found that the intervention had a significant impact (*p* = 0.0002), with one patient in accordance with guidelines out of 73 in the control condition against 29 patients out of 74 in the intervention condition (Table 3). Compared to the control phase, the odds ratio for improved nutritional management for patients included during the intervention phase was OR = 76.8. However, the 95% confidence interval was very large (8.3–713.6) due to the low number of events in the control condition.

We found no influence of the time period or learning effect during the course of the study. In other words, there was an immediate improvement in nutritional management in all clusters.

### 3.3. Evaluation (Nutritional Assessment)

All patients in the intervention group were assessed using at least three of the four diagnosis elements (BMI, weight loss, albumin assay, and MNA), with over 80% having all four available in the medical records. In the control phase, 29% of patients had two elements, almost half (49%) had three, and 15% had all four. BMI was calculated in 100% of patients in both groups (Table 3). The anorexia scale was documented in 92% of patients in the intervention group versus less than 3% in the control group (*p* < 0.0001).

### 3.4. Diagnosis (Nutritional Status)

Where information was available, nutritional status was reassessed using elements of the nutritional assessment. Overall, around 38% of patients had no undernutrition, 40% had moderate undernutrition, and 21% had severe undernutrition. Surprisingly, the reassessed nutritional status matched the status mentioned in the patient record in only 49 patients (33%). For example, 16% of patients with moderate undernutrition and 2% of patients with severe undernutrition had been classified as “no undernutrition”, while 13% of patients with severe undernutrition had been classified as “moderate” (see Table S2). Evaluation of nutritional status was significantly more accurate in the intervention condition compared to control (*p* < 0.0001) but remained imperfect (49% of accurate nutritional assessments in the intervention phase compared to 18% in control) (Table 3).

### 3.5. Prescriptions (According to Nutritional Status)

Prescriptions were significantly more adequate in the intervention phase compared to control, in which only 3 patients out of 73 had prescriptions adapted to their nutritional status. Despite significant improvements in the intervention condition, 43 patients (58%) had inadequate prescriptions with regards to 2006 ESPEN guidelines [23] (Table 3). Among undernourished patients (*n* = 61), only seven (11%) had a completely appropriate treatment overall, and the study showed no significant improvement with the intervention (15% vs. 7%; *p* = 0.4367).

Immuno-modulating supplements were prescribed in the presurgical period (for patients with no or moderate undernutrition) for 94% of patients in the intervention condition versus 71% in the control condition (*p* = 0.0151). For moderately undernourished patients, such supplements were given in the postsurgical period in 47% of patients in the intervention condition versus 10% in the control condition (*p* = 0.0095).

### 3.6. Surgical Outcome

Excluding two patients lost to follow-up before the 30-day period, 38/145 patients (26%) had at least one complication. The majority of these (63%) were of Grade I or II of severity. Postoperative complications were reported more frequently for patients in the intervention phase compared to control (Table 4). Relevant complications (infection, scar disruption, or fistula) affected four patients (28.6%) in the control phase compared to 22 (37.3%) in the intervention phase. However, this difference was not significant (*p* = 0.7576). The rate of reoperation was not significantly different between groups (Table 4). Globally, digestive complications were significantly more frequent in the control phase (85.7%) than in the intervention phase (54.2%; *p* = 0.305).

No significant difference was found when comparing surgical complications in terms of whether nutritional prescriptions were adapted to nutritional status or not (irrespective of intervention or control) (*p* = 0.5426) or whether global management agreed with the guidelines (*p* = 0.5086) (Table S3).

### 3.7. Adverse Events

In total, 194 adverse events were reported during the study, of which 94 (49%) were of low severity, 75 (39%) were moderate, and 25 (13%) were classified as severe. Adverse events were more frequently reported in the intervention condition, in which 58% of patients had at least one adverse event reported (*p* = 0.0500). The majority of these events were of low to moderate severity in both groups and were generally less severe in the intervention group (*p* = 0.0109) (Table S4). Of note, none of these events were considered to be related to the study.

## 4. Discussion

This study aimed to evaluate the impact of a geriatric intervention to improve adherence to international guidelines regarding the screening and management of nutritional risk in older patients undergoing surgery for colorectal cancer. In response to our main objective, we were able to show a significant improvement in adherence to guidelines for the nutritional management of patients. Evaluation, diagnosis, and prescription (according to nutritional status) were considered appropriate and strictly consistent with guidelines in 39.2% of patients in the intervention group compared to only 1.4% in the control group (*p* = 0.0002). The intervention improved the screening and assessment of undernutrition significantly for all criteria except for body mass index, which was already perfectly evaluated in both groups. Prescription of oral nutritional supplements during the perioperative period was also significantly improved (41.9% vs. 4.1%; *p* < 0.0001). However, there were no benefits of the intervention on surgical complications or adverse events.

High levels of evidence support nutritional management as part of multimodal interventions comprising physical activity in addition to perioperative nutritional care [14,18,20]. This should be particularly relevant for frail older adults [34,35], but, in this specific population, the level of evidence remains limited [36]. For interventions in the preoperative period only, the level of evidence is also lower, mainly due to heterogeneity in the multimodal interventions and outcome measures [37,38,39,40]. In a previous single-blind randomized trial of 101 participants, Burden et al. showed that the prescription of oral nutritional supplements (ONS) in the preoperative period was more effective than nutritional advice alone in reducing weight loss and postoperative complications such as infections [41]. Although all recommendations state that patients who have to undergo surgery for digestive cancer should be screened for malnutrition, it is not clear how this will be done [42]. In addition, the added value of immune-modifying supplements compared to classical ONS remains debated [43,44,45,46,47].

The results of studies considering the benefits of immune nutrition on postoperative complications of cancer remain divergent. In their meta-analysis, Hegazi et al. did not find any benefit of immune nutrition compared to standard ONS on infectious or noninfectious complications [45]. More recent meta-analyses have concluded a reduction in infectious complications [48,49]. However, in the meta-analysis by Xu et al., which was focused on colorectal cancer, only studies that used enteral nutrition were included, and it is not possible to extrapolate these results to oral supplementation alone. Moreover, the average age of the patients was much lower than in our study. The ERAS (Enhanced Recovery After Surgery) recommendations also retain the use of ONS in general during the perioperative period [42].

In our study, we showed that the geriatric outreach team could be effective in improving consistency with practice guidelines but in an incomplete way. Implementing change in usual practice is often difficult due to the certain inertia of health systems and entrenched habits of care [50]. The place of geriatric teams could be preponderant in order to improve the care of major geriatric syndromes and, more specifically, undernutrition. Previous studies using geriatric outreach teams or aiming to implement protocols that interfere with current practice have had difficulties showing an impact [51,52]. A recent retrospective study on medical and surgical malnourished patients suggested that embedded nutrition-focused quality improvement programs may improve outcomes such as length of stay or 30-day readmissions, especially for surgical patients [53]. This highlights the key role of the implementation of such programs in the current care context. In addition, we observed that nasogastric tubes for enteral feeding were prescribed less frequently than indications would require. As well as acceptability issues for patients, clinicians themselves might hesitate to put this treatment forward according to their own representations of risk–benefit and acceptability [54].

Other possible obstacles to effective interventions include the insufficient time-lapse for delivering interventions prior to surgery due to surgical schedules and cancer-induced anorexia. Surgical targets in colorectal surgery are usually four weeks from diagnosis to operation. This time-lapse is thought to be sufficient to modify behavior to improve physical function before surgery [55] but may be too short to significantly act on nutritional status. In order to be effective, screening and preventive actions would need to be taken on as soon as possible, ideally from the very first consultation at the surgery clinic. However, in the study by Burden et al., an intake of ONS for only a median of 8 days (5 to 15 days) prior to surgery was sufficient to improve outcomes [41].

Surgical complications and adverse effects, most often of low severity, were more frequently reported during the intervention phase. A plausible explanation for this finding might be a magnified rate of report of adverse events and complications in the intervention group, in which patients benefited from a much closer follow-up. On the other hand, the rate of reoperation was similar in both phases, and the duration of hospitalization appeared reduced by approximately one day during the intervention phase compared to control (Table 2).

The observed inaccuracies in the diagnosis of nutritional status appear to need more questioning. Although the diagnostic accuracy was expectedly improved by the intervention, discrepancies with retrospective diagnoses from the research panel remained for over half of the patients in the intervention group. This suggests that the geriatric teams themselves might have provided flawed recommendations due to an erroneous estimation of the patients’ nutritional status. This might have influenced the results on the main outcome, for which perfect adhesion to guidelines was needed in terms of screening, diagnosis, and management. The implication is that all professionals would benefit from continued education on the subject, including professionals with a priori higher levels of expertise on nutritional prehabilitation. The recent GLIM guidelines for the diagnosis of undernutrition now associate phenotypic and etiologic criteria, in particular related to disease burden and inflammation [56]. Our study shows that the intervention was particularly effective in improving anorexia, with a completion rate of the anorexia scale of 92% in the intervention phase versus only 3% in control.

This study has several strengths. This controlled randomized study was multicentric and evaluated the impact of a perioperative nutritional intervention in various types of hospital settings in the context of current practice, with a particular focus on the at-risk population of older individuals. The number of patients included reached our initial objectives, allowing us to have enough power to make the results reliable. The assessment of undernutrition was holistic and included an evaluation of weight loss as well as a measure of anorexia. The study informs on the generally poor nutritional status of the target population: the prevalence of undernutrition of 61% (21% for severe undernutrition) was greater than previously reported [12]. Although mean BMI, which is known to have rather weak sensitivity, was not reduced, weight loss was reported in over half (57%) of the study population, with a mean weight loss of 8.28% of total body weight in six months. Finally, the level of physical frailty, reflected by a median SPPB score of 8, also highlights a population with a high risk of postsurgical deconditioning and loss of autonomy [57].

This study has some limitations. Firstly, to avoid a risk of contamination, the data were not collected by clinical research assistants on site but by medical teams in each study center, with the result that data were missing concerning the nutritional or frailty status of patients in the control phase. Secondly, due to the missing data, we could only report on the prescription of ONS or immune-modifying supplements but not on their effective consumption by patients, as initially envisaged [26]. Thirdly, information on the duration of postoperative nutrition was lacking. Fourthly, the main outcome included elements of diagnosis and management of undernutrition according to the diagnosis. However, there were frequent discrepancies in both phases between prospective and posthoc diagnosis of severity of undernutrition, which might have influenced the adequacy of nutritional management and generalizability of our results. Finally, as mentioned previously, diagnosis and management guidelines at the time of the study have since been modified. However, this study was primarily aimed at assisting in the implementation and application of recommendations, and the results remain perfectly interpretable in this respect.

In conclusion, our study shows that nutritional diagnosis and management in the perioperative period of CRC in older patients can be sensibly improved by the intervention of a multidisciplinary team, facilitating the adherence and implementation of the recommendations. Further studies would be necessary to assess the lasting effect of this type of intervention, i.e., whether it can lead to a change in team practices over time.

## Figures and Tables

**Table 1:** Number of patients included in each center and each period. The shaded boxes correspond to time periods in intervention.

	Period 1	Period 2	Period 3	Period 4	Period 5	Period 6
Center	8 October 2013–1 April 2014	1 April 2014–1 October 2014	1 October 2014–1 April 2015	1 April 2015–1 October 2015	1 October 2015–1 April 2016	1 April 2016–13 December 2016
1	5	5	3	4	7	5
2	5	6	4	5	5	4
3	4	5	4	3	9	5
4	4	7	1	6	5	6
5	4	6	3	7	5	5

**Table 2:** Patient characteristics at baseline.

		N =	Global	Control	Intervention	*p*-Value ^4^
Median age (min–max)		146	79.6 (55.5–92.8)	79.2 (55.5–89.2)	80.5 (70–92.8)	0.1738 *
Female (%)		147	73 (49.7)	39 (53.4)	34 (45.9)	0.3645
Laboratory results	Median hemoglobin (g/L)	137	11.8 (7–40)	11.5 (7–40)	12 (7.2–16.1)	0.6935 *
	Median albumin (g/L) (min–max)	115	37 (21–48.2)	38 (22–44)	36.9 (21–48.2)	0.7274 *
	Median pre-albumin (g/L) (min–max)	98	0.23 (0.05–3)	0.24 (0.11–1.9)	0.22 (0.05–3)	0.6608 *
	Median CRP (g/L)	94	5.4 (0.4–212)	6.05 (1.1–119)	5.4 (0.4–212)	0.4457 *
Median BMI (Kg/m^2^) (min–max)		146	24.7 (15.8–39.3)	25.3 (17.9–39.3)	24.1 (15.8–38.9)	0.0927
Patients with weight loss (%)		134	76 (56.7)	30 (49.2)	46 (63)	0.1075
Median % weight loss over 6 months (min–max)		50	6.58 (2.06–23.33)	6.56 (2.27–23.33)	6.8 (2.06–20.51)	0.7769 *
Moderate to severe undernutrition (%)		99	61 (61.6)	28 (82.4)	33 (50.8)	0.0022
Cachexia (%)		106	6 (5.7)	1 (2.4)	5 (7.7)	0.4016
Anorexia (%)		93	11 (11.8)	5 (21.7)	6 (8.6)	0.1321 *
Median MNA (min–max) ^1^		59	21.5 (4–26)	21.5 (16–25)	21.8 (4–26)	0.6138 *
Median CIRS-G comorbidity score (min–max)		92	6 (0–19)	6 (1–15)	6 (0-19)	0.8754 *
Number of medications (%)	>6	136	30 (22.1)	18 (29)	12 (16.2)	0.1248
	3–6		73 (53.7)	28 (45.2)	45 (60.8)	
	<3		73 (53.7)	28 (45.2)	45 (60.8)	
Mean fatigue score (SD)		75	6.3 (2.1)	6.3 (2.2)	6.3(2.1)	0.7536 *
Mean handgrip strength (SD) ^2^		65	24.3 (11.4)	21.3 (5.5)	24.5 (11.6)	0.6472
Mean SPPB score (SD) ^3^		75	8.12 (2.87)	7.17 (2.71)	8.2 (2.89)	0.3061 *
Median duration of hospitalization (min–max)		142	10.5 (1–94)	12 (4–94)	9 (1–70)	0.0220 *

BMI: body mass index; CRP: C-reactive protein; CIRS-G: Geriatric Cumulative Illness Rating Scale; MNA: Mini Nutritional Assessment; SPPB: Short Physical Performance Battery; SD: standard deviation. ^1^ Only five measures in control condition; ^2^ only three measures in control condition; ^3^ only six measures in control condition; ^4^ Categorical variables: chi-squared test or Fisher’s exact test (*); continuous variables: Student’s *t*-test or the Wilcoxon test (*).

**Table 3:** Results of the primary outcome and details of its components.

	Control *n* (%)	Intervention *n* (%)	*p*-Value
**Primary outcome**			
Appropriate nutritional management ^1^	1 (1.4)	29 (39.2)	<0.0001
**Mixed model for stepped-wedge design**			
Time from first inclusion in cluster (time-period effect)			0.9897
Time from beginning of intervention phase (learning effect)			0.2498
Control/intervention phase			0.0002
**Details of appropriate nutritional management**			
Screened for under-nutrition			
At least 7 days prior to surgery	11 (15.1)	60 (81.1)	<0.0001
BMI measured	73 (100)	74 (100)	-
Serum albumin measured	52 (71.2)	63 (85.1)	0.0411
Weight loss assessed	60 (82.2)	73 (98.6)	0.0007
MNA performed	14 (19.2)	72 (97.3)	<0.0001
Adequate diagnosis of nutritional status	13 (17.8)	36 (48.6)	<0.0001
Prescriptions in accordance with nutritional status ^2^	3 (4.1)	31 (41.9)	<0.0001

^1^ Appropriate evaluation, diagnosis, and prescription according to nutritional status (primary outcome); ^2^ nutritional status, as assessed retrospectively by the study team.

**Table 4:** Thirty-day postsurgical complications in both groups (N = 145).

		Control *n* (%)	Intervention *n* (%)	*p*-Value ^4^
Patients with at least one postsurgical complication (%)		10 (13.7)	28 (38.9)	0.0006
Mortality (%)		-	4 (6.8)	0.11991
Reoperation (%)		5 (35.7)	9 (15.3)	0.1256 *
Complication type (%)	Fistula/peritonitis	3 (21.4)	8 (14)	0.2563 *
	Hematoma/hemorrhage ^1^	2 (14.3)	11 (19.3)	
	Ileus/occlusion	4 (28.6)	6 (10.5)	
	Infection/sepsis	1 (7.1)	8 (14)	
	Wound dehiscence	1 (7.1)	3 (5.3)	
	diarrhea	1 (7.1)	3 (5.3)	
	Nausea/vomiting		4 (7)	
	Bowel ischemia	1 (7.1)	-	
	General complication or geriatric syndrome ^2^	1 (7.1)	14 (24.6)	
Grade of complications ^3^ (%)	I	3 (21.4)	25 (42.4)	0.0321 *
	II	4 (28.6)	19 (32.2)	
	IIIa	5 (35.7)	3 (5.1)	
	IIIb	2 (14.3)	8 (13.6)	
	IV	-	-	
	V	-	4 (6.8)	

^1^ These were: anemia (x8), rectorrhagia or melena (x4), and perihepatic hematoma; ^2^ these were: pain (x3), non-specific fever (x3), delirium (x2), pneumonia, acute kidney injury, hypokalemia, urinary retention, oral fungus, and hypertension. ^3^ According to the Common Terminology Criteria for Adverse Events (CTCAE) of the National Cancer Institute and the Clavien–Dindo classification [33]. ^4^ Categorical variables: chi-squared test or Fisher’s exact test (*); continuous variables: Student’s *t*-test or the Wilcoxon test (*).

## Supplementary Materials

The following are available online at https://www.mdpi.com/article/10.3390/nu13072347/s1, Table S1: Cancer localization and surgery type; Table S2: Detail of observed discrepancies between reported and calculated nutritional status for incorrectly assessed patients, Table S3A,B: Rate of 30-day surgical complications according to adequate/inadequate nutritional management (A) and according to compliance/non-compliance with guidelines of overall management (evaluation and management) (B); Table S4: Adverse events (AE) reported during the study.

## ANC Working Group

Gilles Albrand, Mustapha Adham, Sophie Armand, Hélène Astrieud, Saida Azzaoui-Cuzon, Patrick Bachmann, Xavier Barth, Emilie Berthoux, Colette Blanc, Damien Blehaut, Isabelle Bonnefoy, Laure Bonnet, Cécile Bouix, Raphaël Bourdariat, Eléonore Brocard, Elisabeth Castel-Kremer, Brigitte Comte, Eddy Cotte, Dominique Delaunay, Filbert Djeudi, Sylvie Do-Adro, Aurélien Dupré, Michel El-Bechwaty, Claire Falandry, Chantal Fingal, Yves François, Viktoria Frebault, Béatrice Galamand, Vincent Garbit, Olivier Gléhen, Nicolas Golse, Sandrine Guillemin, Patrice Guyot, Max Haine, Johannes Hartwig, Jérémy Hermann, Sophie Humblot, Anne Humeau, Vahan Kepenekian, Céline Leclerc, Gaëlle Leguludec, Murielle Lemoine, Catherine Lombard, Jean-Yves Mabrut, Alain Marchand, Maud Marchand, Fatima Marini, Delphine Mazard, Pierre Meeus, Pascale Méré, Nathalie Michel-Laengh, François Mithieux, Olivier Monneuse, Agnès Montpied, Justine Ondet, Audrey Pasquion, Guillaume Passot, Elisa Pelascini, Denis Péré-Vergé, Hervé Perrier, Sylvie Perrin, Patrice Peyrat, Marie Poiblanc, Gilles Poncet, Bénédicte Prost, Nathalie Rançon, Olivier Raspado, Michel Rivoire, Monique Roldan, Pascale Roux, William Sebag, Martine Serrano, Gaëtan Singier, Dominique Stalet, Catherine Terret, Patrick Van Box Som, Delphine Vaudoyer, Françoise Veyri.
