# Supplementary material for: Impact of a Geriatric Intervention to Improve Screening and Management of Undernutrition in Older Patients Undergoing Surgery for Colorectal Cancer: Results of the ANC Stepped-Wedge Trial
Source: Nutrients. 2021 Jul 9;13(7):2347. doi: 10.3390/nu13072347 (PMC8308889; doi:10.3390/nu13072347)
Supplement: Supplementary file 1 [file nutrients-13-02347-s001.zip › nutrients-1260389-supplementary.pdf]

Supplementary Material

Table S1. Cancer localization and surgery type.

N=147	Global	Control	Intervention	P-value
**Cancer Localization**				
Caecum	5 (3.4)	4 (5.5)	1 (1.4)	0.7564*
Ascending colon	51 (34.7)	27 (37)	24 (32.4)	
Transverse colon	1 (0.7)	1 (1.4)	-	
Descending colon	17 (11.6)	8 (11)	9 (12.2)	
Sigmoid colon	15 (10.2)	9 (12.3)	6 (8.1)	
Colon and other[1]	4 (2.7)	2 (2.7)	2 (2.7)	
Colon not specified	14 (9.5)	6 (8.2)	8 (10.8)	
Rectum	37 (25.2)	16 (21.9)	21 (28.4)	
Rectum and other[2]	1 (0.7)		1 (1.4)	
Caecum and rectum	1 (0.7)		1 (1.4)	
Not specified	1 (0.7)		1 (1.4)	
**Surgery performed**				
Right hemicolectomy	56 (38.1)	29 (39.7)	27 (36.5)	Not calculable
Left hemicolectomy	18 (12.2)	10 (13.7)	8 (10.8)	
Sigmoid colon resection	12 (8.2)	8 (11)	4 (5.4)	
Segmental colectomy	2 (1.4)	-	2 (2.7)	
Total or subtotal colectomy	8 (5.4)	6 (8.2)	2 (2.7)	
Anterior rectum resection	11 (7.5)	4 (5.5)	7 (9.5)	
Abdomino-perineal rectum resection	1 (0.7)	1 (1.4)	-	
Abdomino-perineal amputation	5 (3.4)	2 (2.7)	3 (4.1)	
Total proctectomy	12 (8.2)	5 (6.8)	7 (9.5)	
Rectum resection, not specified	5 (3.4)	2 (2.7)	3 (4.1)	
Complex surgery[1,2]	6 (4.1)	5 (6.8)	7 (9.5)	
Not specified	11 (7.5)	1 (1.4)	10 (13.5)	
**Surgery Method**				
Open surgery	22 (15)	13 (17.8)	9 (12.2)	0.2092
Mini-invasive (laparoscopy and/or robot assisted)	48 (32.7)	19 (26)	29 (39.2)	
Not specified	77 (52.4)	41 (56.2)	36 (48.6)	

[1] these were: (colon+ liver), (colon + stomach), (colon + stomach + gall bladder), (colon + prostate + gall bladder); [2] these were: (rectum+ small intestine + bladder resection). P-value : Categorical variables Chi-squared test or Fisher's exact test (*) - Continuous variables Student t-test or the Wilcoxon test (*).

Table S2 – Detail of observed discrepancies between reported and calculated nutritional status for incorrectly assessed patients (n=98)[a].

Reported Nutritional Status	Calculated Nutritional Status	Frequency	Percentage
No under-nutrition	Moderate	16	16.3
	Severe	2	2.0
	NC	24	24.5
Moderate	No under-nutrition	3	3.1
	Severe	13	13.3
	NC	6	6.1
Severe	No under-nutrition	1	1.0
	Moderate	4	4.1
	NC	1	1.0
Other	NC	1	1.0
Not Reported	No under-nutrition	4	4.1
	Moderate	7	7.1
	NC	16	16.3

Note. NC= not calculable; a. For 98 patients out of 147 (66.7%), nutritional status was estimated inadequately.

Table S3 A & B – Rate of 30-day surgical complications according to adequate/inadequate nutritional management (A) and according to compliance/non-compliance with guidelines of overall management (evaluation and management) (B), N=145.

**A**	**Global**	**Adequate prescription n(%)**	**Inadequate prescription n(%)**	**P-Value**
Patients with at least one post-surgical complication	38 (26.2)	10 (30.3)	28 (25)	0.5426

**B**	**Global**	**Conformity of management with guidelines n(%)**	**Non-conformity n(%)**	**P-Value**
Patients with at least one post-surgical complication	38 (26.2)	9 (31)	29 (25)	0.5086

Table S4 – Adverse Events (AE) reported during the study

		Control n(%)	Intervention n(%)	P-Value
**Patients with at least one AE (%)**		31 (41.9)	44 (57.9)	0.0500
Severity of AE	Low	16 (32)	78 (54.2)	0.0109
	Moderate	28 (56)	47 (32.6)	
	Severe	6 (12)	19 (13.2)	
